# Conserved Outer Tegument Component UL11 from Herpes Simplex Virus 1 Is an Intrinsically Disordered, RNA-Binding Protein

**DOI:** 10.1128/mBio.00810-20

**Published:** 2020-05-05

**Authors:** Claire M. Metrick, Andrea L. Koenigsberg, Ekaterina E. Heldwein

**Affiliations:** a Department of Molecular Biology and Microbiology, Tufts University School of Medicine, Boston, Massachusetts, USA; b Graduate Program in Biochemistry, Tufts School of Graduate Biomedical Sciences, Tufts University School of Medicine, Boston, Massachusetts, USA; c Graduate Program in Molecular Microbiology, Tufts School of Graduate Biomedical Sciences, Tufts University School of Medicine, Boston, Massachusetts, USA; Princeton University

**Keywords:** herpesvirus, tegument, viral protein, viral assembly, intrinsically disordered protein (IDP), RNA-binding protein, small‐angle X‐ray scattering (SAXS), conformational flexibility, structural model, liquid-liquid phase separation (LLPS), biomolecular condensate

## Abstract

A distinguishing morphological feature of all herpesviruses is the multiprotein tegument layer located between the nucleocapsid and lipid envelope of the virion. Tegument proteins play multiple roles in viral replication, including viral assembly, but we do not yet understand their individual functions or how the tegument is assembled and organized. UL11, the smallest tegument protein, is important for several distinct processes in replication, including efficient virion morphogenesis and cell-cell spread. However, the mechanistic understanding of its role in these and other processes is limited in part by the scant knowledge of its biochemical and structural properties. Here, we report that UL11 from herpes simplex virus 1 (HSV-1) is an intrinsically disordered, conformationally dynamic protein that undergoes liquid-liquid phase separation (LLPS) *in vitro*. Intrinsic disorder may underlie the ability of UL11 to exert multiple functions and bind multiple partners. Sequence analysis suggests that not only all UL11 homologs but also all HSV-1 tegument proteins contain intrinsically disordered regions of different lengths. The presence of intrinsic disorder, and potentially, the ability to form LLPS, may thus be a common feature of the tegument proteins. We hypothesize that tegument assembly may involve the formation of a biomolecular condensate, driven by the heterogeneous mixture of intrinsically disordered tegument proteins.

## Abstract (precis)

Herpesvirus virions contain a unique tegument layer sandwiched between the capsid and lipid envelope and composed of multiple copies of about two dozen viral proteins. However, little is known about the structure of the tegument or how it is assembled. Here, we show that a conserved tegument protein UL11 from herpes simplex virus 1, a prototypical alphaherpesvirus, is an intrinsically disordered protein that undergoes liquid-liquid phase separation *in vitro*. Through sequence analysis, we find intrinsically disordered regions of different lengths in all HSV-1 tegument proteins. We hypothesize that intrinsic disorder is a common characteristic of tegument proteins and propose a new model of tegument as a biomolecular condensate.

## INTRODUCTION

Herpesviruses are large enveloped viruses that infect nearly all species of vertebrates and some invertebrates. In humans, these viruses cause lifelong infections that are generally asymptomatic or cause mild symptoms, such as orofacial lesions (cold sores) and genital lesions, but can also cause severe disease in certain subpopulations. Nine human herpesviruses belong to three subfamilies, alpha-, beta-, and gammaherpesviruses, that differ in tropism and pathogenesis yet share many conserved replication mechanisms, including viral assembly and morphogenesis.

Herpesvirus virions are composed of an icosahedral capsid encasing the double-stranded DNA (dsDNA) genome and are coated with lipid envelopes containing viral glycoproteins. Between the capsid and the envelope lies the tegument layer that is unique to herpesviruses and is a dense network of dozens of viral proteins that is structurally maintained even following removal of the viral envelope (1). In the prototypical alphaherpesvirus herpes simplex virus 1 (HSV-1), the tegument contains multiple copies of 24 viral proteins and several host proteins (2). Tegument proteins are assigned to either the inner layer or the outer layer depending on their association with the capsid or viral envelope, respectively (3). While the inner tegument layer is anchored on the nucleocapsid, and the viral proteins in this layer are present in stoichiometric quantities (4), the outer tegument layer is more heterogeneous and appears amorphous (5). The assembly of the tegument onto the capsids occurs largely in the cytoplasm during the process of secondary envelopment wherein capsids gain their external envelope by budding into host vesicles derived from the Golgi/endosomal network (6, 7). Many tegument proteins are found associated with these vesicles in infected cells and are thought to regulate the secondary envelopment process by a mechanism yet to be identified (reviewed in reference 3). In addition to their importance in virion structure and morphogenesis, many tegument proteins perform other functions. For example, inner tegument protein UL37 is thought to bridge the viral capsid and envelope by binding capsid-associated UL36 and membrane-anchored glycoprotein K (gK) and UL20 (8). UL37 may also target capsids to the correct site of secondary envelopment, based on the structural similarities between CATCHR proteins and UL37 from HSV-1 and pseudorabies virus (PRV) (9, 10). Different tegument proteins with known structural roles also have regulatory functions, including modulation of host intrinsic and innate immune responses, upregulation of viral gene expression, downregulation of host gene expression, and capsid trafficking through the cytoplasm (reviewed in references 3, 11, and 12).

**FIG 1:**
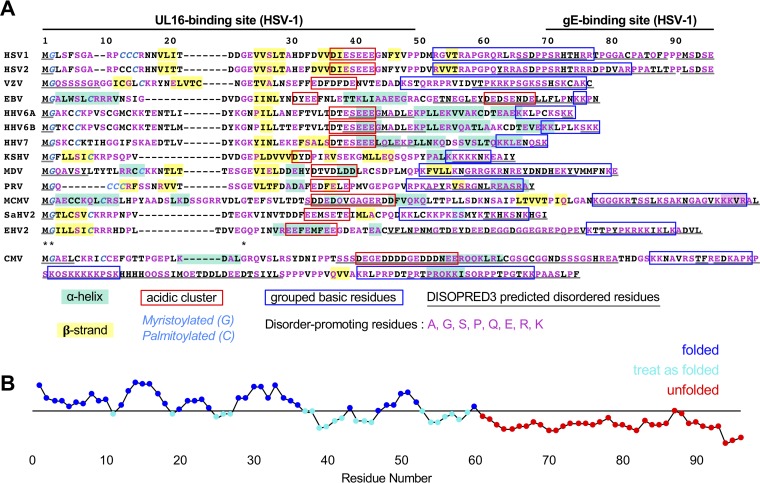
Alignment and characteristics of UL11 homolog sequences. (A) Sequences of UL11 homologs from herpesviruses aligned to HSV-1 UL11 with HSV-1 residue numbers marked. Human virus sequences used include HSV-1 strain 17 UL11 (RefSeq accession no. YP_009137085.1), HSV-2 strain HG52 UL11 (RefSeq YP_009137162.1), VZV strain Dumas ORF49 (RefSeq NP_040171.1), EBV strain B95-8 BBLF1 (RefSeq YP_401686.1), CMV strain AD169 UL99 (RefSeq P13200.3), HHV-6A strain Uganda-1102 U71 (RefSeq NP_042964.1), HHV-6B strain Z29 U71 (RefSeq NP_050250.1), HHV-7 strain JI U71 (RefSeq YP_073811.1), and KSHV strain GK18 ORF38 (RefSeq YP_001129391.1). Other representative animal virus sequences used include Marek’s disease virus (MDV) strain Md5 UL11 (RefSeq YP_001033939.1), pseudorabies virus (PRV) composite strain UL11 (RefSeq YP_068364.1), murine cytomegalovirus (MCMV) strain Smith UL99 (RefSeq YP_214100.1), saimiriine herpesvirus 2 (SaHV-2) ORF38 (RefSeq NP_040240.1), and equine herpesvirus 2 (EHV-2) strain 86/67 myristoylated tegument protein (RefSeq NP_042635.1). All sequences show the NCBI reference sequence accession number or code in parentheses. Conserved residues are marked with an asterisk. Myristoylated glycines and palmitoylated cysteines, experimentally determined or predicted, are shown in italicized type in cyan text. Acidic clusters, experimentally defined or predicted, are boxed in red. Groups of basic residues are boxed in blue. Beta strands and alpha helices predicted by PSIPRED (http://bioinf.cs.ucl.ac.uk/psipred/) are highlighted in yellow and light teal, respectively. Disorder-promoting residues (A/G/S/P/Q/E/R/K) are colored in magenta. Residues predicted to be disordered by DISOPRED3 (http://bioinf.cs.ucl.ac.uk/psipred/) are underlined in gray. (B) Representation of disorder by residue in HSV-1 UL11 as predicted by FoldUnfold (http://bioinfo.protres.ru/ogu/). Residues predicted to be in natively folded regions are shown in blue, and residues predicted to be in unfolded regions are shown in red. Residues scoring below the threshold (black line) but surrounded by folded residues are to be treated as folded and are shown in cyan.

The UL11 gene is conserved across all herpesvirus subfamilies (Fig. 1A) and encodes a small, membrane-associated outer tegument protein that localizes primarily to the Golgi (13). UL11 homologs from HSV-1, herpes simplex virus 2 (HSV-2), Epstein-Barr virus (EBV), cytomegalovirus (CMV), and likely all others are N-terminally myristoylated (13–21). UL11 homologs from HSV-1, EBV, and likely, HSV-2 are also palmitoylated (17, 22–24) (Fig. 1A). These two modifications enable UL11 to bind to cytoplasmic membranes (13, 14, 22) and associate with detergent-resistant membrane microdomains, or lipid rafts (23, 24). Palmitoylation is also required for both Golgi targeting and strong membrane interactions (22). Surprisingly, myristoylation and palmitoylation are not essential for replication because UL11 mutants lacking these modifications can partially rescue replication-deficient UL11-null HSV-1 (25).

In many herpesviruses, UL11 homologs are important for efficient viral replication, and their deletion results in reductions in viral titer that range in magnitude depending on the virus (15, 25–29). Deletion of UL11 homologs causes accumulation of nonenveloped capsids in the cytoplasm (29–32). Thus, UL11 is thought to participate in secondary capsid envelopment through an unclear mechanism (3). UL11 binds capsid-associated tegument protein UL16 (33–36) using a cluster of acidic residues within its N terminus (19, 27, 37, 38) (Fig. 1A). This conserved interaction may help bridge the capsid and envelope during secondary envelopment (3). In addition to its role in secondary envelopment, UL11 appears to have other functions. In alphaherpesviruses, UL11 forms a ternary complex with UL16 and UL21 (39) that binds the cytoplasmic tail of glycoprotein E (gE) (40) (Fig. 1A) and modulates its function in cell-cell fusion (39). Finally, UL11 localizes to the nucleus (41) for reasons that are unclear.

Despite the importance of UL11 in multiple viral processes, sparse biochemical and structural information limits both our mechanistic understanding of its roles in viral replication and tegument assembly. Here, we employed circular dichroism (CD), limited proteolysis, small-angle X-ray scattering (SAXS), and light microscopy to characterize the structure and solution properties of HSV-1 UL11. We report that UL11 is an intrinsically disordered protein (IDP) that undergoes liquid-liquid phase separation (LLPS) *in vitro*. We propose that intrinsic disorder underlies the ability of UL11 to exert multiple functions and bind multiple partners, which include UL16, gE, and potentially, RNA. Sequence analysis highlights that all UL11 homologs and, more broadly, all HSV-1 tegument proteins contain intrinsically disordered regions of different lengths, suggesting that the presence of disorder is a common feature of the tegument. Based on the ability of heterogeneous mixtures of intrinsically disordered proteins to assemble into biological condensates *in vivo*, we hypothesize that formation of similar assemblies by outer tegument proteins at the sites of secondary envelopment may contribute to viral morphogenesis.

## RESULTS

### Expression and purification of UL11.

**FIG 2:**
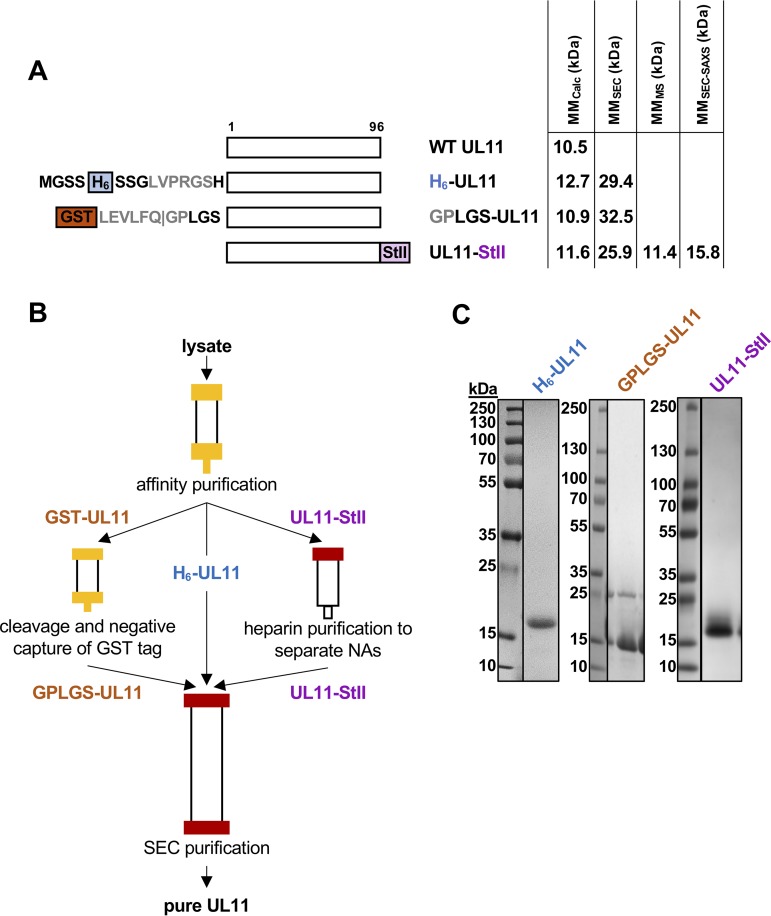
Expression and purification of UL11 constructs. (A) Schematic representation of UL11 sequence and UL11 constructs used in this work. Protease recognition sites are shown in gray. Construct names and molecular masses calculated from the sequence (MM_Calc_) and determined experimentally (this paper) (by SEC, MS, and SEC-SAXS) are shown. WT, wild type; H_6_, His_6_ tag; GST, glutathione *S*-transferase tag; StII, Strep-tag II. (B) Purification scheme for UL11 constructs. (C) Coomassie blue-stained gels of purified UL11 constructs.

To characterize the structure and biochemical properties of HSV-1 UL11, we expressed several constructs in Escherichia coli (Fig. 2A) and purified them (Fig. 2B and C). UL11 containing N-terminal His_6_ tag (H_6_-UL11) was purified in soluble form (Fig. 2C), but the yield was low. To increase expression and to allow removal of the affinity tag, an N-terminal glutathione *S*-transferase (GST) tag followed by an HRV3C (PreScission) protease cleavage site was added, generating the GST-UL11 construct. Cleavage with PreScission protease yielded UL11 with an N-terminal GPLGS linker sequence (Fig. 2A). The GST-UL11 construct had higher protein yields but could not be purified to homogeneity (Fig. 2C). We then sought an alternative affinity tag that would not attract nonspecific contaminants. Therefore, a small, C-terminal Strep-tag II (StII) was introduced to improve both the yield and purity (UL11-StII) (Fig. 2A). We placed the new tag at the C terminus, away from the predicted functional sites (Fig. 1A) or any predicted structure at the N terminus (Fig. 1B). This construct had a higher yield and could be purified to homogeneity (Fig. 2C). UL11 lacks tryptophans and has only one tyrosine, which complicates concentration estimates based on absorption at 280 nm. Addition of the StII tag (WSHPQFEK), which contains a tryptophan, also improved spectrophotometric tracking. Since all constructs displayed similar characteristics once purified, the UL11-StII construct was used in the majority of subsequent studies.

### UL11 copurifies with E. coli RNA.

**FIG 3:**
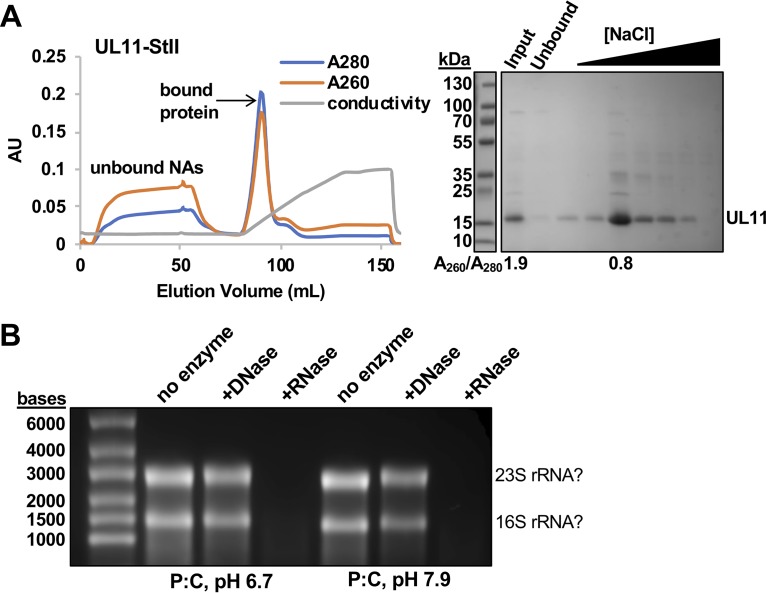
UL11 copurifies with RNA. (A) Fast protein liquid chromatography (FPLC) shows that UL11-StII is separated from copurifying nucleic acids on heparin resin. Protein, but not nucleic acids (NAs), binds heparin resin and elutes with a salt gradient (conductivity). UL11-StII is present in the eluted fraction, but not in the unbound fraction. The *y* axis shows absorbance or conductivity (in arbitrary units [AU]). Samples were resolved by SDS-PAGE and stained with Coomassie blue. The *A*_260_/*A*_280_ ratio shown below the SDS-PAGE confirms the presence of nucleic acids in the input fraction but not in the eluted fraction. (B) Nucleic acids that copurify with UL11-StII in acidic or neutral phenol-chloroform (P:C) are susceptible to digestion by RNase, but not DNase. The banding pattern is characteristic of E. coli rRNA, as marked. Samples were resolved on a formaldehyde agarose gel and stained with ethidium bromide. The gel in panel A was split to remove unrelated lanes, but contrast settings remain consistent between related gels.

During initial purification of UL11-StII, the eluate from streptactin affinity resin appeared pure by sodium dodecyl sulfate-polyacrylamide gel electrophoresis (SDS-PAGE). However, the *A*_260_/*A*_280_ ratio of 1.9 indicated the presence of approximately 60% nucleic acids (NAs) (95) (Fig. 3A). To identify which NAs copurified with UL11, we used a nuclease digestion assay. After streptactin affinity purification, bound NAs were isolated from UL11/NA complex samples using phenol-chloroform extraction and incubated with DNase or RNase or left untreated. UL11-bound NAs were sensitive to RNase but not DNase treatment (Fig. 3B). Furthermore, the size and banding pattern of the UL11-bound NAs were consistent with rRNA (96). Whether UL11 binds rRNA with some degree of specificity or copurifies with rRNA because these are the major RNA species in E. coli remains unclear. In any case, our results suggest that UL11 binds not only UL16 and gE but also some RNA species. The addition of a heparin affinity step (Fig. 3A) to the UL11-StII purification scheme (Fig. 2B) to separate UL11 from copurifying NAs reduced the *A*_260_/*A*_280_ ratio to 0.8, which corresponds to 2% NA content (Fig. 3A). All further experiments with UL11-StII in this study were performed with samples free of bound NAs (“NA-free”).

### UL11 displays hallmarks of an intrinsically disordered protein.

**FIG 4:**
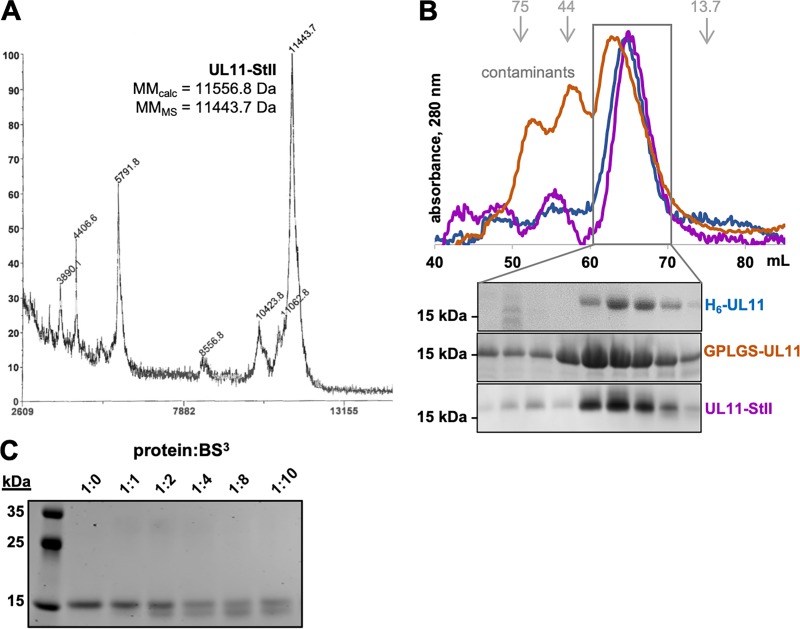
UL11 is an elongated monomer. (A) MALDI-TOF spectrum of UL11-StII. The *x* axis shows the mass-to-charge ratio (*m/z*) in daltons and the *y* axis shows relative abundance (%) scaled for the mass range in *x*. (B) Size exclusion chromatography (SEC) *A*_280_ traces and gels. The elution volumes of calibration standards used to calculate apparent molecular masses (kDa) are depicted in gray and with gray arrows. (C) Coomassie blue-strained gel of UL11 incubated with increasing amounts of BS^3^ cross-linker.

Despite the calculated molecular masses ranging from 10.9 to 12.7 kDa (Fig. 2A), UL11 constructs consistently migrated as 15- to 20-kDa species on SDS-polyacrylamide gels (SDS-PAGs) (Fig. 2C). Slow electrophoretic mobility is typically observed for proteins that are posttranslationally modified (44), but E. coli-expressed UL11 is unlipidated because prokaryotes lack endogenous *N*-myristoyltransferase activity (45). Moreover, matrix-assisted laser desorption ionization−time of flight mass spectrometry (MALDI-TOF MS) analysis of UL11-StII indicated the molecular mass of 11.4 kDa (Fig. 2A and 4A). Abnormal electrophoretic mobility, manifesting as a 1.2- to 1.8-times-higher apparent molecular mass than expected from the sequence, is also observed for intrinsically disordered proteins (IDPs) (46, 47; reviewed in references 48 and 49). IDPs are thought to bind less SDS than globular proteins due to a distinct sequence composition, e.g., high net charge and low hydrophobicity, which would cause them to migrate more slowly than expected (reviewed in reference 48). Higher proline content, typical of IDPs, increases protein rigidity, which further reduces electrophoretic mobility (reviewed in reference 49).

During purification, all three UL11 constructs eluted from size exclusion chromatography (SEC) at a volume consistent with that of an ∼30-kDa globular protein, much larger than the molecular mass predicted from the UL11 sequence (Fig. 4B). This observation held true not only for H_6_-UL11 and GPLGS-UL11, which potentially contained copurifying NAs, but also for NA-free UL11-StII (Fig. 4B). IDPs typically have reduced SEC mobility due to reduced protein compaction (reviewed in reference 49). However, SEC cannot distinguish an elongated monomer from an oligomer. To probe the oligomeric state, NA-free UL11-StII was cross-linked with bis(sulfosuccinimidyl)suberate (BS^3^), a homobifunctional cross-linking reagent that reacts with primary amines. In the presence of increasing amounts of cross-linker, no bands of higher molecular mass were detected on SDS-PAGs (Fig. 4C). Instead, cross-linked UL11-StII migrated faster (Fig. 4C) probably due to the presence of intramolecular cross-links that prevented complete unfolding under SDS-PAGE conditions. Thus, UL11 is monomeric in solution under these experimental conditions.

Sequence analysis of HSV-1 UL11 identified several features characteristic of disorder-containing proteins (reviewed in reference 49). First, the amino acid sequence is enriched in disorder-promoting residues P, G, E, A, S, R, K, and Q (50) (56% total residues) and depleted of order-promoting residues W, Y, F, C, I, L, and N (50) (19% total residues) (Fig. 1A). The low content of aromatic residues in particular results in a very low extinction coefficient at 280 nm (51), 1,490 M^−1 ^cm^−1^ for HSV-1 UL11. Second, HSV-1 UL11 has little predicted secondary structure (∼15% β strand and no α helices) (Fig. 1A); a predicted disordered C terminus, residues 61 to 96, as estimated by FoldUnfold server (Fig. 1B); and no predicted globular domains, according to the IUPred2A server. Finally, it has a hypervariable sequence such that in the Pfam database (42), the UL11 family is represented only by residues 1 to 39, and even these do not align well across UL11 homologs (Fig. 1A). Collectively, these features, along with aberrant electrophoretic and chromatographic mobility, suggested the presence of structural disorder in UL11.

### UL11 undergoes LLPS *in vitro*.

**FIG 5:**
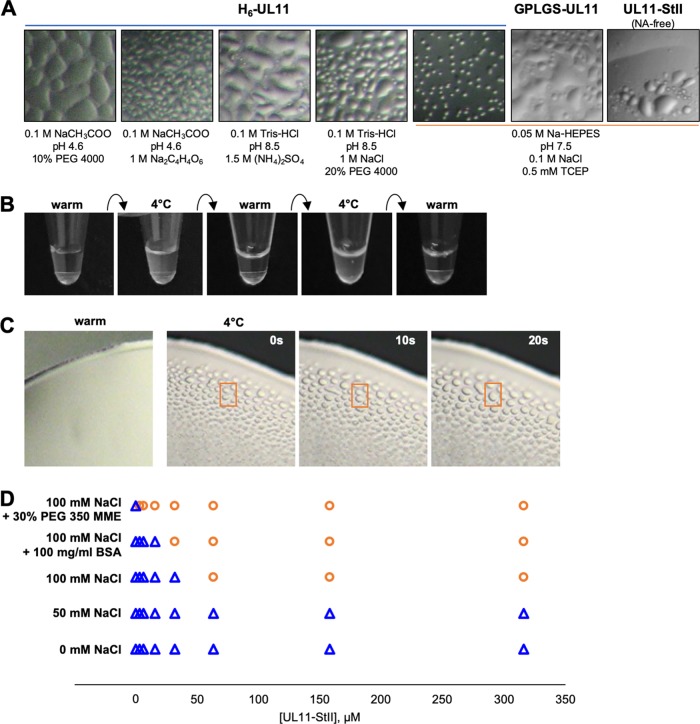
UL11 undergoes liquid-liquid phase separation. (A) Microscopic images of UL11 constructs in solution show representative images of liquid-liquid phase separation (LLPS) seen under a variety of conditions, including purification buffer and for H_6_-UL11, crystallization screens (1:1 mix of protein-solution in vapor diffusion chamber). (B) Macroscopic images of UL11-StII in solution in purification buffer show reversibility of UL11-StII phase separation (cloudiness) upon temperature cycling. (C) Time lapse microscopic images of UL11-StII condensates in purification buffer display hallmark liquid-like properties of surface wetting and droplet merging (selected example in orange box). (D) LLPS (separation, orange circles; clear solution, blue triangles) occurs with increasing protein concentration, increasing salt concentration, and increasing molecular crowding.

During affinity purification of H_6_-UL11 at 4°C, the elution fraction appeared cloudy, yet no pellet formed after centrifugation. Furthermore, after SEC at 4°C, the most concentrated fractions of the UL11-containing peak also appeared cloudy. Under the microscope, such cloudy samples contained droplets reminiscent of liquid-liquid phase separation (LLPS) (Fig. 5A). This phenomenon was observed not only in the purification buffer but also with a wide range of additives, including ∼1/3 of 752 sitting-drop crystal screening conditions (Fig. 5A). Similar droplet formation was observed with GPLGS-UL11 and UL11-StII samples (Fig. 5A).

A common property of LLPS is its temperature-dependent reversibility (43, 52). Purified NA-free UL11-StII samples appeared turbid when cooled down to 4°C but became clear when warmed up by hand. Subsequent cooling to 4°C restored turbidity (Fig. 5B). Observing these warmed and cooled samples of UL11-StII under a light microscope confirmed that turbid samples had droplets whereas clear samples did not. Furthermore, these droplets displayed the characteristic liquid-like properties of merging and surface wetting (Fig. 5C). The ability of UL11 to undergo LLPS was also dependent on protein concentration, salt concentration, and the presence of molecular crowders polyethylene glycol 350 monomethyl ether (PEG 350 MME) and bovine serum albumin (BSA) (Fig. 5D). Notably, the addition of molecular crowders to the most concentrated UL11 samples at room temperature immediately prompted LLPS formation, which manifested as turbidity. We hypothesize that LLPS is an intrinsic property of UL11 because all constructs manifested LLPS regardless of the presence of copurifying NAs, the identity of the affinity tag, or its placement.

### UL11 is mostly disordered in solution.

**FIG 6:**
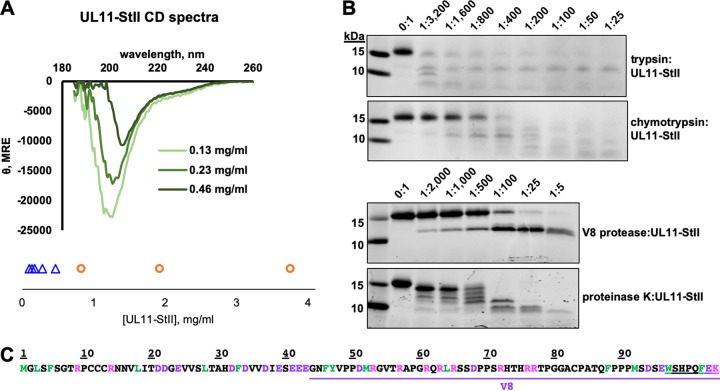
UL11 lacks defined secondary structure and is sensitive to proteolysis. (A) CD spectra of UL11-StII at several concentrations show primarily random-coil characteristics. UL11-StII undergoes LLPS (LLPS, orange circles; clear solution, blue triangles) with increasing concentration in CD buffer at ambient temperature. See also Table 1. (B) Limited proteolysis of UL11-StII using trypsin, chymotrypsin, V8 protease, or proteinase K with increasing amounts of protease. (C) Sequence of UL11-StII with potential proteolysis sites colored (green, chymotrypsin; pink, trypsin; purple, V8). V8 fragment identified by MALDI-TOF mass spectrometry and N-terminal sequencing (see Fig. S1 in the supplemental material) is underlined in purple. The StII sequence is double underlined.

HSV-1 UL11 has little predicted secondary structure, ∼15% β strand and no α helices (Fig. 1A). To assess the secondary structure of NA-free UL11-StII, we used far-UV circular dichroism (CD) at three concentrations (Fig. 6A). The CD spectrum of UL11-StII at the lowest concentration resembled the spectrum of a random-coil polypeptide, with a large negative molar ellipticity near 200 nm and a negative shoulder in the 220- to 240-nm region. Such spectra are typical of IDPs (reviewed in reference 49). Thus, UL11 is largely disordered in solution. Unexpectedly, with increasing concentration, the absolute magnitude of the CD signal decreased (Fig. 6A), which is often associated with protein aggregation, although no precipitation was observed in the samples. Instead, increasing LLPS was seen with increasing concentration of UL11 (Fig. 6A), and this phenomenon occurred at a lower concentration of UL11 in CD buffer relative to purification buffer. While droplet formation was not obvious under the light microscope at the UL11 concentrations used in the CD experiment, the droplets could have been too small to detect by eye. Thus, LLPS could, in principle, account for the decrease in absolute magnitude of the CD signal, as has been reported for phase-separating proline-arginine-rich peptides (52). Although the CD spectra of UL11-StII had random-coil characteristics at higher protein concentrations, the large negative peak shifted toward a higher wavelength (Fig. 6A). The CDSSTR algorithm (53) as implemented in Dichroweb estimated that the proportion of the regularly structured elements (including regular α helices and regular β strands) increased from 13% to 23% with increasing protein concentration (Table 1). This suggested that some regular secondary structural elements within UL11 may form as the concentration increases.

**TABLE 1:** CD structural parameters

UL11 concn (mg/ml)	Reference set or parameter^*a*^	NRMSD^*b*^	% of residues^*c*^					
			Helix1	Helix2	Strand1	Strand2	Turns	Unordered
0.46	SP175	0.043	0.08	0.12	0.12	0.11	0.17	0.4
	Set 6	0.056	0.03	0.04	0.21	0.09	0.17	0.46
	Set 7	0.041	0.06	0.1	0.19	0.09	0.21	0.35
	Avg		0.06	0.09	0.17	0.10	0.18	0.40

0.23	SP175	0.034	0.01	0.07	0.17	0.13	0.16	0.46
	Set 6	0.023	0.04	0.03	0.13	0.08	0.13	0.59
	Set 7	0.024	0.02	0.04	0.12	0.07	0.14	0.58
	Avg		0.02	0.05	0.14	0.09	0.14	0.54

0.13	SP175	0.019	0.02	0.06	0.15	0.12	0.17	0.49
	Set 6	0.022	0.02	0.04	0.11	0.07	0.12	0.64
	Set 7	0.023	0.02	0.04	0.07	0.05	0.1	0.72
	Avg		0.02	0.05	0.11	0.08	0.13	0.62

a The reference set is the set of curves used in CDSSTR (53) to estimate secondary structure content from experimental curves.

b NRMSD, normalized root mean square deviation between calculated and experimental CD spectra.

c The percentage of residues in a regular helix (93) (Helix1), distorted helix (Helix2), regular strand (Strand1), distorted strand (Strand2), and beta turns (Turns) and the percentage of residues lacking secondary structure (Helix1, Helix2, Strand1, Strand2, and Turns) (Unordered).

10.1128/mBio.00810-20.1 FIG S1 Identification of V8 proteolytic fragments. (A) Limited proteolysis of UL11-StII with V8 protease. N-terminal sequence of the major proteolytic fragment is shown along with the molecular masses calculated from the sequence (MM_calc_) and determined by mass spectrometry (MM_MS_). (B) MALDI-TOF mass spectrometry analysis of the same digest. The x axis shows mass to charge ratio (m/z) in Da and the y axis shows relative abundance (%) scaled for the mass range in x. Molecular mass (MM_MS_) of the major peak is shown along with the mass calculated from the sequence (MM_calc_). Download FIG S1, PDF file, 1.5 MB. Copyright © 2020 Metrick et al. 2020 Metrick et al. This content is distributed under the terms of the Creative Commons Attribution 4.0 International license.

To further probe the disordered nature of UL11, we subjected NA-free UL11-StII to limited proteolysis with a panel of proteases either preferentially targeting specific residues—basic (trypsin), acidic (V8), or bulky hydrophobic (chymotrypsin) residues—or having low specificity (proteinase K). Only digestion with V8 resulted in a proteolytically resistant fragment (Fig. 6B and C). This fragment was identified by N-terminal sequencing and MALDI-TOF MS (see Fig. S1 in the supplemental material) and reflects the locations of the cleavage sites. Such high proteolytic susceptibility suggests a lack of structured regions to protect against cleavage, i.e., the presence of intrinsic disorder.

### UL11 is an extended, conformationally dynamic molecule.

**FIG 7:**
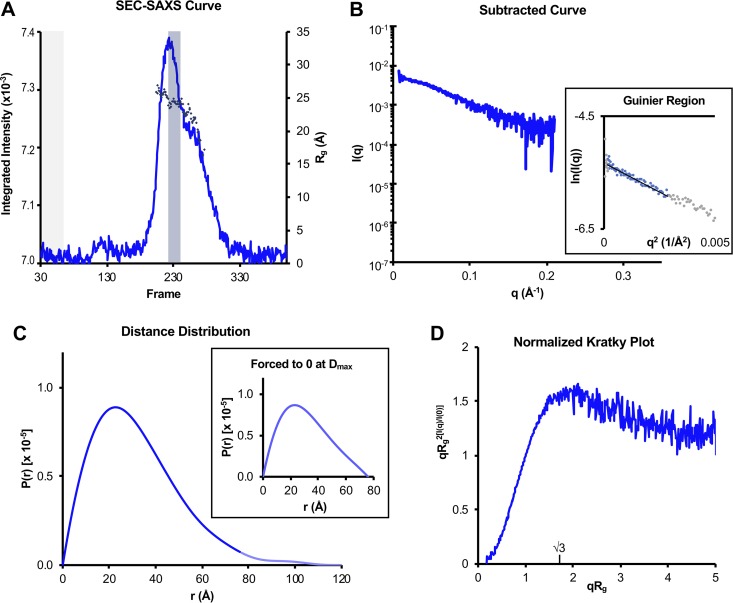
SAXS analysis of UL11. (A) Trace of integrated scattering intensities for each frame collected in the UL11-StII SEC-SAXS experiment. Buffer curves (frames shaded gray) were averaged and subtracted from each scattering curve to calculate *R_g_* (diamonds) frame by frame. Data from curves giving a consistent *R_g_* within the major SEC peak (frames shaded blue) were averaged, and the buffer average was subtracted from this sample average to give the subtracted SAXS curve in panel B. (B) Subtracted SAXS curve for UL11-StII with Guinier plots (inset, including linear fits) at the low-angle region (*qRg *< 1.3). These regions were selected with AUTORG such that residuals were evenly distributed around zero. Data points in gray were excluded from the Guinier analysis. (C) Pair distance distribution functions [*P*(*r*)] for UL11-StII calculated from scattering curves in panel B, comparing curves calculated with *D*_max_ forced to zero (inset) or not forced to zero (main panel). In the main panel, the faded blue line shows the continuation of the UL11 *P*(*r*) after the *D*_max_ when forced to zero. (D) Normalized (dimensionless) Kratky plot was calculated from scattering curve in panel B. Parameters from these data are also summarized in Table 2. Data for UL11-StII have been deposited into the Small Angle Scattering Biological Data Bank (SASBDB) under code SASDEX4.

To characterize the conformational state and flexibility of UL11 in solution, we performed small-angle X-ray scattering coupled with size exclusion chromatography (SEC-SAXS). SEC-SAXS profiles were collected for NA-free UL11-StII (Fig. 7A), and SAXS data were processed by averaging frames with a consistent radius of gyration (*R_g_*) value and subtracting averaged buffer frames to generate subtracted curves (Fig. 7B). These data were selected only from frames corresponding to the SEC peak to limit analysis to homogeneous UL11-StII. Parameters calculated from subtracted SAXS data are summarized in Table 2.

**TABLE 2:** SAXS structural parameters

Parameter	Value(s) for UL11-StII
Guinier analysis	
*I*(0) (cm^−1^)^*a*^	0.00477 ± 0.000041
*R_g_* (Å)^*b*^	24.29 ± 2.0
*q*_min_ (Å^−1^)^*c*^	0.01254
*qR_g_* max^*d*^	1.30239
Coefficient of correlation, *R*^2^^*e*^	0.938

*P*(*r*) analysis (forced to 0/not forced)^*f*^	
*I*(0) (cm^−1^)	0.004748 ± 0.00003566/0.004866 ± 0.00009504
*R_g_* (Å)	24.74 ± 0.1599/26.29 ± 1.712
*D*_max_ (Å)^*g*^	76/120
*q* range (Å^−1^)^*h*^	0.0074 − 0.2802/0.0074 − 0.2802
χ^2^ (total estimate from GNOM)^*i*^	1.0385 (0.8640)/0.9935 (0.7203)

AMBIMETER analysis of *P*(*r*) (forced to 0/not forced)^*f*^	
Compatible shape categories^*j*^	720/153
Ambiguity score^*k*^	2.857/2.185
Uniqueness^*l*^	Highly ambiguous/might be ambiguous

a *I*(0), scattering intensity at zero angle.

b *R_g_*, radius of gyration.

c *q*_min_, lowest *q* value used in the Guinier analysis.

d *qR_g_* max, maximum *q* value used in the Guinier analysis × *R*_g_.

e *R*^2^, linear fit of the Guinier plot.

f (forced to 0/not forced) = *P*(*r*) forced to 0 at *D*_max_ in DATGNOM (91)/*P*(*r*) not forced to 0.

g *D*_max_, maximum dimension calculated from *P*(*r*).

h *q* range, *q* values used in *P*(*r*) plot.

i χ^2^ fit of *P*(*r*) to data. The total quality estimate from GNOM (91) is shown in the parentheses.

j The number of shape topologies, *M*, compatible with *P*(*r*) evaluated by AMBIMETER (94).

k The ambiguity score is log *M* and correlates with probability of finding a false-positive result in a three-dimensional (3D) reconstruction (94).

l Uniqueness of 3D reconstruction of *P*(*r*) evaluated by AMBIMETER (94).

The molecular mass for UL11-StII calculated by the volume of correlation method (54), 15.8 kDa, fell between the expected values for a monomer and a dimer (Fig. 2A and Table 2). Although no oligomeric species were observed in the cross-linking experiments (Fig. 4C), these experiments were done at a lower protein concentration than that used in the SEC-SAXS experiment. Unfortunately, concentration-independent methods currently in use cannot accurately estimate the molecular mass of a disordered protein from SEC-SAXS curves. A Guinier plot was assembled from the subtracted SAXS curve (Fig. 7B), and the linear region was used to calculate the *R_g_* of the sample (Table 2). Linearity in the Guinier region (*qR_g_* >1.3) additionally indicates a lack of intermolecular attraction (aggregation) or repulsion that would interfere with parameter calculation. We hypothesize that UL11-StII is monomeric under the conditions of SEC-SAXS experiment but cannot rule out the presence of higher-molecular mass species.

To calculate more accurate values of *R_g_* and the maximum dimension (*D*_max_) of UL11-StII, a pair distance distribution function [*P*(*r*)] was generated from the subtracted curve (Table 2). The *P*(*r*) derived from UL11-StII SAXS data was asymmetrical and trailed off to large distances (Fig. 7C). This profile was observed regardless of whether the function was preemptively forced to zero or not, and is characteristic of an elongated and/or flexible molecule (Fig. 7C) (55, 56). Thus, assuming the sample was fully monomeric, UL11 adopts an extended conformation, with *R_g_* = 24.7 Å and *D*_max_ = 76 Å.

To estimate conformational flexibility, a dimensionless Kratky plot was calculated (56) (Fig. 7D). In contrast to the expected parabolic shape associated with a globular, ordered protein (54), the UL11-StII Kratky plot remained elevated in value after the *qR_g_* = √3 inflection point (Fig. 7D), which is characteristic of IDPs (56, 57).

### Modeling UL11 as a conformational ensemble.

Due to disorder, IDPs sample multiple conformations in solution and cannot be modeled as single conformers using *ab initio* bead modeling. Indeed, AMBIMETER reported hundreds of compatible shape categories for UL11-StII (Table 2). Instead, it is more appropriate to model IDPs as conformational ensembles—groupings of multiple static structures that represent possible conformations (reviewed in reference 49). Conformational ensembles do not provide a unique description of the conformer population because any given IDP likely exists as a very large number of conformers. Instead, they provide possible, nonunique solutions consistent with the experimental data that are useful for representing the conformational diversity of an IDP under study.

To generate conformational ensembles for UL11, we used ensemble optimization modeling (EOM), a method commonly used to model SAXS data for conformationally dynamic systems (58, 59). EOM quantifies flexibility of the system using the *R*_flex_ parameter, where *R*_flex_ is 0% for a fully rigid system and 100% for a fully flexible system (59). The *R*_flex_ of a random pool representing 10,000 possible conformations is usually ∼85 to 90%. For a rigid system, e.g., a macromolecule that exists as a small number of conformers, *R*_flex_ would be expected to decrease significantly after ensemble optimization modeling. However, for UL11-StII, there was no appreciable decrease in the *R*_flex_ between the random pool and optimized ensembles of models representing the SAXS data (Table 3), which indicates that UL11 is highly flexible and exists as multiple conformers of different shape.

**TABLE 3:** Dimensional and flexibility characteristics of optimized ensembles^*a*^

Pool or ensemble	Parameter or conformational state	*R*_g_ (Å)^*b*^	*D*_max_ (Å)^*c*^	Proportion (%)^*d*^	*R*_flex_ (%)^*e*^	χ^2^^*f*^
Random pool^*g*^	***Avg***	***27.16***	***82.86***	***100***	***86.57***	

Optimized ensemble 1^*h*^	Compact^*i*^	20.75	59.61	44.4		
	Intermediate^*j*^	27.35	81.195	44.4		
	Extended^*k*^	35.95	111.43	11.1		
	***Avg***	***25.48***	***74.24***	***100***	***83.96***	***0.983***

Optimized ensemble 2	Compact	21.38	61.47	54.6		
	Intermediate	28.59	79.97	36.4		
	Extended	37.55	109.55	9.1		
	***Avg***	***25.44***	***72.2***	***100***	***84.97***	***0.983***

Optimized ensemble 3	Compact	21.94	63.63	55.5		
	Intermediate	27.78	81.53	33.3		
	Extended	35.79	107.3	11.1		
	***Avg***	***25.55***	***75.43***	***100***	***84.30***	***0.983***

Avg ensemble	Compact	21.36	61.57	51.5		
	Intermediate	27.91	80.90	38.0		
	Extended	36.43	109.43	10.4		

a The average values for the random pool or ensembles are shown in italic boldface type.

b *R_g_*, radius of gyration of structural models.

c *D*_max_, maximum dimension of structural models.

d Proportion is the percentage of curves representing each category within the pool or ensemble.

e *R*_flex_ is a measurement of information entropy, correlated with conformational flexibility, in the pool or ensemble. *R*_flex_ would be 100% for a totally flexible system versus 0% for an ideal rigid system.

f χ^2^, measurement of fit between the SAXS curve generated from the optimized ensemble of models and experimental data.

g The random pool is 10,000 structural models representing UL11-StII generated by RANCH within EOM 2.0 (58, 59).

h Optimized ensembles 1, 2, and 3 are from the random pool that together represent the experimental data as chosen by GAJOE within EOM 2.0 (58, 59).

i Compact, category of models with *R_g_ *< 24 and *D*_max_ < 71.

j Intermediate, category of models with 26 < *R_g_ *< 31 and 77 < *D*_max_ < 83.

k Extended, category of models with *R_g_ *> 35 and *D*_max_ > 107.

**FIG 8:**
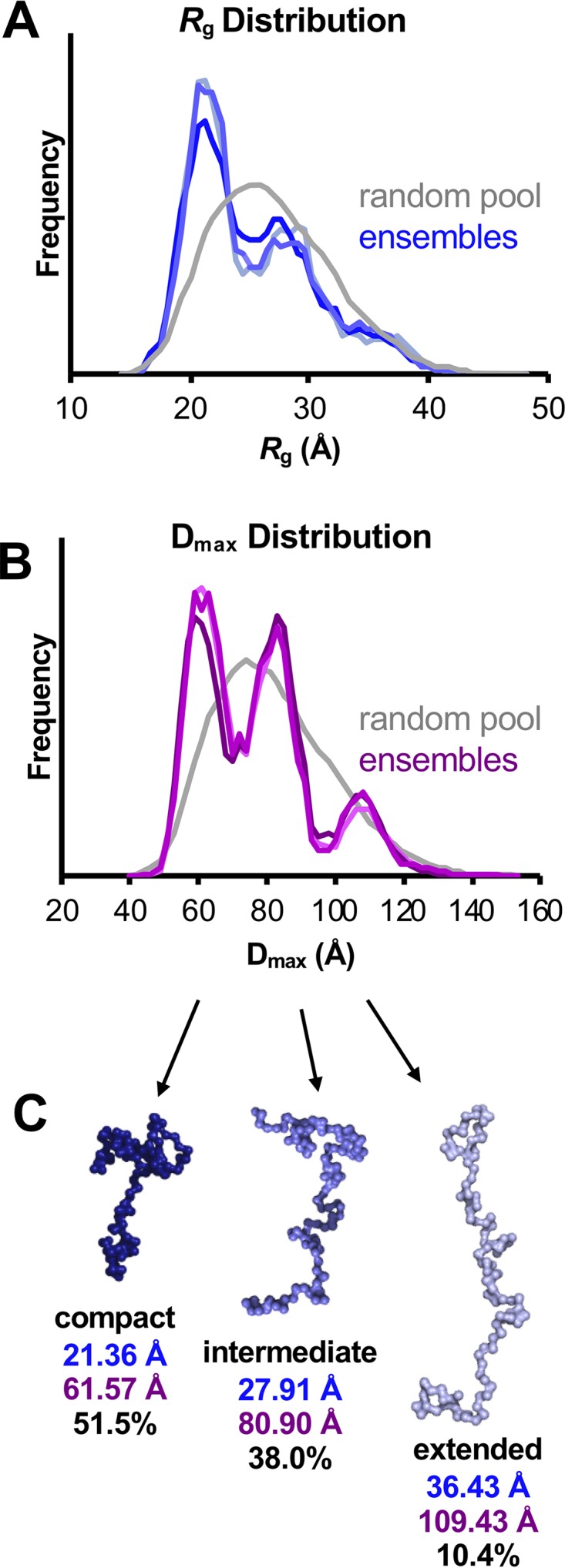
Ensemble optimization modeling (EOM) of UL11-StII. (A and B) Histograms comparing the distribution of *R_g_* values (A) and *D*_max_ values (B) between a pool of 10,000 randomly generated structures (gray) and three independently optimized representative ensembles of structures (blues in panel A; purples in panel B) indicate the presence of three conformations. (C) Representative structures of the conformations found in the optimized ensembles are shown with the average *R_g_* (blue) and *D*_max_ (purple) values and the percentage of the ensemble they represent. These models suggest possible behavior of the flexible protein in solution, but they do not represent the only solution. These values are also summarized in Table 3.

EOM generates probability density distributions, which represent the probability that the protein adopts a certain conformation, and the relative ratios of those conformations. Three independently optimized conformational ensembles representing UL11-StII identified three populations in the histogram: compact (∼50%), intermediate (∼40%), and extended (∼10%) (Fig. 8 and Table 3), which define three major conformational ensembles of UL11-StII. Overall, SAXS measurements suggest that UL11 samples multiple conformations in solution.

## DISCUSSION

In addition to being structural components of the herpesviral virions, tegument proteins perform numerous functions during herpesvirus replication: they regulate viral morphogenesis, mediate intracellular capsid trafficking, control gene expression, and suppress the host antiviral response, to mention a few. Our limited mechanistic understanding of their roles in viral replication would greatly benefit from knowing the structures and the biochemical properties of tegument proteins. However, structures of only 6 out of 24 HSV-1 tegument proteins are available (10, 60–65), and structural information on tegument proteins in other herpesviruses is even scarcer. Expanding our structural knowledge of the tegument would inform not only mechanistic studies of individual tegument proteins but also models of how they are arranged within the tegument layer.

Here, we investigated the biochemical properties, solution behavior, and structural characteristics of HSV-1 UL11, the smallest conserved tegument protein that is important for efficient virion morphogenesis and cell-cell spread. We show that recombinant, unlipidated HSV-1 UL11 is a conformationally dynamic, intrinsically disordered protein (IDP). Several characteristics of disorder-containing proteins were evident from sequence analysis: high content of disorder-promoting residues along with low content of order-promoting residues, a hypervariable sequence across homologs, little predicted secondary structure, and no predicted globular domains. Experimentally, the intrinsically disordered nature of UL11 was evident from the predominantly random-coil character of its CD spectrum, high proteolytic sensitivity, reduced electrophoretic and chromatographic mobility, and IDP signatures within the SAXS spectra (reviewed in references 56 and 57).

All experiments reported here used E. coli-expressed unlipidated UL11, whereas in HSV-1-infected cells or in uninfected cells overexpressing UL11, UL11 is both myristoylated and palmitoylated (13–15, 22, 23). Nevertheless, we propose that the IDP characteristics of UL11 observed *in vitro* reflect its behavior *in vivo*. For example, UL11 homologs isolated from transfected and infected cells migrate with a higher apparent molecular mass on SDS-PAGs (14, 27, 66) similarly to E. coli-expressed unlipidated UL11. While slower electrophoretic mobility was previously attributed to posttranslational modifications (14, 27, 66), one report showed that unlipidated HSV-1 UL11 migrated just as slowly as the lipidated UL11 (22). Therefore, we propose that the slow electrophoretic mobility of UL11 *in vivo* is instead due to the intrinsic disorder. Another *in vivo* phenotype that could be attributed to the intrinsic disorder is that in the absence of lipid modifications or the binding partner UL16, UL11 and its homologs are relatively unstable in cells, presumably due to rapid turnover (17, 25, 67, 68). Such behavior is typical of IDPs because they are more susceptible to proteolysis than globular proteins. Association with a binding partner such as UL16 or a membrane could potentially protect UL11 from proteolysis and increase its lifetime.

UL11 homologs have hypervariable sequences even within the N-terminal UL11 Pfam motif (Fig. 1A). Nonetheless, UL11 homologs share conserved functional motifs within their N-terminal portions, such as lipidation sites and acidic clusters. The UL11/UL16 interaction has been demonstrated for their homologs from HSV-1 (19), PRV (19), CMV (68), and Kaposi’s sarcoma herpesvirus (28), and thus, the UL16-binding site located within the UL11 N terminus (37) is likely conserved. Even the more divergent C-terminal portions share certain sequence characteristics (overabundance of disorder-promoting residues and basic residues) and may thus share conserved functions, e.g., the ability to bind gE (67) or negatively charged binding partners such as nucleic acids (this paper). These sequence characteristics are not limited to UL11 homologs from human herpesviruses but extend to animal herpesviruses from all three subfamilies (Fig. 1A).

We found that E. coli-produced UL11 bound endogenous RNA, potentially through interactions with its basic C terminus. Overabundance of basic residues is frequently found in low-complexity sequences where they are thought to promote disorder (48), and as a result, many proteins containing basic intrinsically disordered regions (IDRs) bind NAs either specifically or promiscuously (69). The banding pattern of the observed RNA was consistent with rRNA (96). rRNA makes up a significant portion of the total E. coli RNA pool (70), so a UL11/rRNA interaction could be nonspecific. However, another HSV-1 tegument protein, UL21, which also copurifies with E. coli RNA, does not show any preference for binding rRNA (61). UL11 could, thus, bind rRNA with some degree of specificity. Many other tegument proteins bind RNA (61, 71), so future studies should explore RNA as a potential UL11-binding partner and the effect of RNA binding by UL11 on UL11/gE interaction (40).

Ensemble modeling of SAXS data suggests that despite its highly dynamic nature, UL11 samples three distinct conformational states: extended, intermediate, and compact. Transient compaction of UL11 between these distinct states or simply within its flexible landscape of conformations could be driven by intramolecular interactions, for example, electrostatic interactions between the N-terminal acidic cluster and the basic residues within the C terminus. Although we did not see any evidence of oligomerization at lower concentrations, we could not rule out the presence of higher-molecular-mass UL11 species under the conditions of the SEC-SAXS experiment. Therefore, one or more of the observed conformational states could correspond to a UL11 oligomer. Regardless, the ability to adopt multiple conformations could allow UL11 to take on many functions, with each conformation mediating distinct functions and interactions, such as binding to protein partner UL16 or gE, which, in turn, may stabilize particular conformations. Binding different RNA species could also induce or stabilize a particular structure of UL11, as has been observed with HIV Tat (reviewed in reference 69), increasing the conformational and thus functional landscape available to UL11. Finally, a conformational shift from unstructured to more structured could occur at higher UL11 concentrations, e.g., in our CD (Fig. 6A and Table 1) and SAXS (Fig. 7 and 8) experiments or in lipid rafts (24).

Many IDPs and IDR-containing proteins can undergo phase separation not only *in vitro* (72) but also *in vivo*, forming biomolecular condensates also known as proteinaceous membrane-less organelles (PMLOs) (73). For example, FUS, an RNA-binding protein associated with several neurodegenerative diseases, forms “spherical droplets” in the neuronal nuclei, while its recombinant, purified form forms similar phase-separated “liquid droplets” *in vitro* (74). Biomolecular condensates are heterogeneous mixtures enriched in IDPs and IDR-containing proteins that engage in many multivalent specific and nonspecific interactions to ensure that condensates remain stable even when individual components are lost (72). UL11 readily undergoes phase separation *in vitro* (this work) and may do so *in vivo*, judging by reports that it localizes to punctate cytosolic structures (19, 38) and nuclear speckles (41). Phase separation is influenced by many factors, including protein concentration, salt concentration, binding partners, and other solution components (43, 52; reviewed in references 75 and 76). Although *in vitro*, the UL11 condensates primarily formed in cold conditions and dissipated before reaching physiological temperature, in the presence of molecular crowders, concentrated UL11 underwent phase separation at room temperature. Thus, specific conditions may promote formation of UL11 biomolecular condensates *in vivo*, for example, binding to gE, UL16, or RNA or clustering within lipid rafts (24). Reevaluating UL11 puncta formed *in vivo* in the context of biomolecular condensates will be important for elucidating the mechanisms of phase separation by UL11 *in vivo* and their dependence on binding partners.

**FIG 9:**
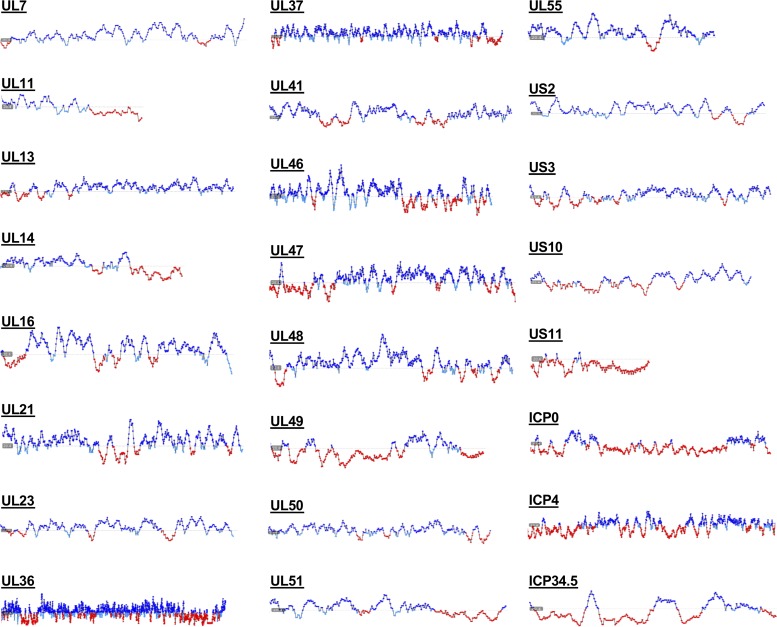
Predicted disorder in HSV-1 tegument proteins. Predicted flexibility of HSV-1 tegument proteins as calculated by FoldUnfold (http://bioinfo.protres.ru/ogu/). Folded residues are indicated in blue, while unfolded residues are indicated in red. Protein length is not shown to scale.

Along with other tegument proteins, UL11 is important for virion assembly during capsid budding at the cytoplasmic membranes. However, how the tegument assembles around the capsid and how the tegument proteins are arranged within the tegument layer are unclear. It is tempting to speculate that rather than forming an ordered structure, the outer tegument exists as a biomolecular condensate, a heterogeneous milieu composed of many partially disordered molecules that make multiple redundant nonspecific interactions. Indeed, outer tegument proteins create a complex network that has both specific (3) and nonspecific interactions that may be important for tegument formation (6). In fact, several outer tegument proteins, including UL21 (61) and UL48 (63), have been reported to contain short or long IDRs, and our analysis suggests that all 24 HSV-1 tegument proteins contain disordered regions of variable lengths (Fig. 9). A biomolecular condensate model of the outer tegument could explain why deletions of individual tegument proteins typically do not disrupt the tegument layer and cause only modest defects in viral replication. Puzzling reports that virions missing individual outer tegument proteins have increased amounts of other tegument proteins, RNA, or even host proteins (2, 77) can be explained by the latter serving as “space fillers” within the biological condensate of the tegument. The ability of the outer tegument to form a biomolecular condensate, rather than a strictly ordered protein network, would allow for productive replication even when the availability of one or more outer tegument proteins fluctuates. Furthermore, based on their liquid-like characteristics, phase-separated droplets are spherical (reviewed in reference 72). Formation of such phase-separated droplets, driven by membrane-associated tegument proteins such as UL11, could promote budding during secondary envelopment by causing the spherical bud to pinch off into *trans*-Golgi network (TGN)-derived membranes, akin to a lava lamp. Such a budding mechanism would not require the capsid, which is consistent with the formation of capsid-less L particles during normal HSV-1 infection (1). Indeed, phase separation has recently been suggested as a mechanistic mediator of membrane deformation during endocytosis (78). Furthermore, viral inclusions formed during replication and assembly of viruses such as rabies virus (79) and influenza virus (80) share characteristics of membrane-less organelles. Finally, it has been proposed that herpesviral replication compartments in the nucleus—the sites of viral gene expression and replication—may be formed by a mechanism that involves LLPS (81). We hypothesize that secondary envelopment is a new example of the use of LLPS in herpesviral replication. While this proposed mechanism does not rule out contributions of host factors or undiscovered viral mediators of membrane deformation and scission, it would ensure envelopment regardless of whether these mediators are present. Future studies of the tegument structure and assembly mechanisms should, thus, take into account the presence of structural disorder in tegument proteins.

## MATERIALS AND METHODS

### Sequences and analyses.

Sequences for UL11 homologs from eight human herpesviruses: herpes simplex virus 1 (HSV-1) strain 17 UL11 (RefSeq accession no. YP_009137085.1), HSV-2 strain HG52 UL11 (RefSeq YP_009137162.1), vesicular stomatitis virus (VSV) strain Dumas ORF49 (RefSeq NP_040171.1), Epstein-Barr virus (EBV) strain B95-8 BBLF1 (RefSeq YP_401686.1), cytomegalovirus (CMV) strain AD169 UL99 (RefSeq P13200.3), human herpesvirus 6A (HHV-6A) strain Uganda-1102 U71 (RefSeq NP_042964.1), HHV-6B strain Z29 U71 (RefSeq NP_050250.1), HHV-7 strain JI U71 (RefSeq YP_073811.1), Kaposi’s sarcoma herpesvirus (KSHV) strain GK18 ORF38 (RefSeq YP_001129391.1), Marek's disease virus (MDV) strain Md5 UL11 (RefSeq YP_001033939.1), pseudorabies virus (PRV) composite strain UL11 (RefSeq YP_068364.1), murine cytomegalovirus (MCMV) strain Smith UL99 (RefSeq YP_214100.1), saimiriine herpesvirus 2 (SaHV-2) ORF38 (RefSeq NP_040240.1), and equine herpesvirus 2 (EHV-2) strain 86/67 myristoylated tegument protein (RefSeq NP_042635.1) were aligned in Clustal Omega (http://www.ebi.ac.uk/Tools/msa/clustalo/) and adjusted manually. ExPASy ProtParam (http://web.expasy.org/protparam/) was used to calculate protein molecular masses, amino acid content, and extinction coefficients. CSSpalm (http://csspalm.biocuckoo.org/online.php) was used to predict palmitoylation sites. Myristylation was marked on glycine 2 based on previous studies (13–15) and conservation. JPred (82) (http://www.compbio.dundee.ac.uk/jpred/) and PsiPred (83) (bioinf.cs.ucl.ac.uk/psipred) were used to predict secondary structure. FoldUnfold (84) (http://bioinfo.protres.ru/ogu/) was used with default settings to predict disorder. IUPred2A (85) (https://iupred2a.elte.hu/) was used with default settings to predict structured domains.

### Cloning and expression constructs.

A plasmid encoding full-length UL11 from HSV-1 strain 17 preceded by a His_6_ tag and a thrombin cleavage site (H_6_-UL11) in a pET28 vector was a gift from John Wills (Pennsylvania State University). This plasmid contains an A8T mutation in UL11, but because this region of protein is poorly conserved (Fig. 1A), the mutation was not considered problematic. Furthermore, UL11 with this mutation successfully rescues a UL11-null virus (25). UL11 was amplified from the pET28 vector using primers CM121 and CM122 (see Table S1 in the supplemental material), digested with NcoI and XhoI, and subcloned into the NcoI/XhoI-digested pGEX-6P-1 backbone to produce plasmid pCM054 which encodes UL11 preceded by a glutathione *S*-transferase (GST) tag, a human rhinovirus (HRV) 3C (PreScission) protease site, and a Leu-Gly-Ser linker, GST-HRV3C-UL11. To add a C-terminal Strep-tag II (StII), single overlap extension (SOE) PCR was performed on pCM054 using primers pGEX3, pGEX5, CM131, and CM132 (Table S1). The insert was digested with NcoI and XhoI and subcloned into the NcoI/XhoI-digested pCM054 vector to produce plasmid pCM056 encoding GST-HRV3C-UL11-StII. Finally, the same insert was subcloned into the original pET-28 vector, again using NcoI and XhoI, to produce plasmid pCM055 encoding UL11-StII.

10.1128/mBio.00810-20.2 TABLE S1 Primers used to generate UL11 constructs. Download Table S1, PDF file, 0.03 MB. Copyright © 2020 Metrick et al. 2020 Metrick et al. This content is distributed under the terms of the Creative Commons Attribution 4.0 International license.

### Recombinant protein expression and purification.

H_6_-UL11 was expressed in E. coli strain T7 cells in LB for 4 to 8 h at 37°C using induction with 1 mM isopropyl-β-d-thiogalactopyranoside (IPTG) at an optical density at 600 nm (OD_600_) of 0.6 to 1.0. GST-HRV3C-UL11 was expressed in E. coli strain Rosetta (Novagen) cells using an autoinduction protocol (86). Briefly, a small culture of cells, grown overnight at 37°C in terrific broth (TB) with 1% glucose and 2 mM MgSO_4_, was inoculated at a ratio of 1:100 into TB supplemented with 0.2% lactose and 2 mM MgSO_4_ and grown for 4 h at 37°C and then for 18 to 22 h at 25°C. StII-tagged constructs were expressed in E. coli strain Rosetta or LOBSTR (87) cells at 37°C using IPTG induction as described above.

Cells were harvested by centrifugation at 5,000 × *g* for 30 min at 4°C, resuspended in buffer A [50 mM HEPES, 100 mM NaCl, 0.5 mM tris(2-carboxyethyl)phosphine (TCEP) (pH 7.5)] with added 0.1 mM phenylmethanesulfonylfluoride (PMSF), and 1× cOmplete protease inhibitor cocktail (Roche), and lysed using a Microfluidizer. After removal of insoluble material from lysates by centrifugation at 20,000 × *g* for 30 min at 4°C, each construct was purified using affinity and size exclusion chromatography in buffer A as follows. H_6_-UL11 was captured on Ni Sepharose 6 Fast Flow (GE Healthcare Life Sciences), washed sequentially with 30 mM and 50 mM imidazole, and eluted with 300 mM imidazole in buffer A. GST-HRV3C-UL11 was captured on glutathione Sepharose 4B (GE Healthcare Life Sciences) and eluted with 10 mM reduced glutathione in buffer A. The GST tag was removed from GST-HRV3C-UL11 to make GPLGS-UL11 (where GPLGS is a linker) by incubating the protein with a recombinant, GST-tagged PreScission protease at a 1:30 protease/protein molar ratio overnight at 4°C during dialysis against 50 to 100 volumes of buffer A to remove reduced glutathione. Uncleaved GST-HRV3C-UL11, excess GST tag, and PreScission protease were removed from GPLGS-UL11 either by passing the mixture over a standalone glutathione column and collecting the flowthrough or by including glutathione Sepharose Fast Flow HiTrap columns (GSTrap FF; GE Healthcare Life Sciences) in line with size exclusion chromatography (below). Prior to purification, UL11-StII-containing lysates were supplemented with small amounts of egg white avidin (Sigma), and protein was captured on Strep-Tactin Sepharose resin (GE Healthcare Life Sciences) and eluted with 5 mM *d-*desthiobiotin (Sigma) in buffer A. UL11-StII was subsequently separated from copurifying nucleic acids using a heparin Sepharose HiTrap column (GE Healthcare Life Sciences) and eluted with a gradient of 0.1 to 1.0 M NaCl in buffer A.

All constructs were further purified by size exclusion chromatography using Superdex 75 (GE Healthcare Life Sciences) in buffer A. The column was calibrated using a Gel Filtration Calibration kit containing blue dextran, aldolase, conalbumin, ovalbumin, and RNase A (GE Healthcare Life Sciences). Calibration curves were generated to calculate the apparent molecular weight and Stoke’s radius of UL11 according to manufacturer’s instructions and using the formula *K*_av_ = (*V_e_* − *V_o_*)/(*V_c_* − *V_o_*) and is proportional to log molecular mass, where *K*_av_ is the partition coefficient, *V_e_* is the elution volume, *V_c_* is the geometric column volume, and *V_o_* is the void volume. *K*_av_ is proportional to the log of molecular mass.

Throughout purification, UL11 samples were concentrated in Amicon Ultra centrifugal concentrators (Millipore) with a 10-kDa molecular weight cutoff and stored with 0.1 mM PMSF and 1× Halt protease inhibitor cocktail (Pierce). Final samples were evaluated for sample purity and concentration using SDS-PAGE and spectrophotometrically using the following extinction coefficients and molecular masses: H_6_-UL11 (1,490 M^−1 ^cm^−1^ and 12,679.96 Da), GPLGS-UL11 (1,490 M^−1 ^cm^−1^ and 10,928.10 Da), and UL11-StII (6,990 M^−1 ^cm^−1^ and 11,556.79 Da), as calculated using the ProtParam utility in the ExPASy suite (https://web.expasy.org/protparam/). It should be noted that both H_6_-UL11 and the cleaved product of GST-HRV3C-UL11 lack tryptophans, which results in less reliable estimates of concentration based on absorbance at 280 nm. In contrast, the addition of the Strep-II affinity tag to UL11-StII provided a tryptophan residue for more reliable concentration determination based on spectroscopic measurements.

### Nuclease digestion.

Nuclease digestion assays were performed as described previously (61). Briefly, after streptactin resin purification, copurifying endogenous E. coli nucleic acids (NAs) in complex with UL11-StII were extracted by phenol-chloroform precipitation. Either slightly acidic phenol-chloroform to preferentially isolate RNA (phenol-chloroform-isoamyl alcohol [25:24:1] [pH 6.7]; Fisher Scientific) or slightly basic phenol-chloroform (phenol-chloroform-isoamyl alcohol [25:24:1] [pH 7.9]; Ambion) were mixed with aliquots of UL11-StII-NA complex in a 1:1 volume ratio and centrifuged at 16,000 × *g* and ambient temperature for 5 min. The upper aqueous layer was removed, added to 10 μg glycogen (Life Technologies), 1/10 volume of 3 M sodium acetate, and 10 volumes of isopropanol (where “volume” is the original volume of the aliquot), and incubated for 10 min at –80°C. Pellets containing nucleic acids were collected by centrifugation at 16,000 × *g* for 20 min at 4°C and washed with 75% ethanol prior to resuspension in water for analysis. Nucleic acid samples were digested in 1× Turbo DNase buffer with either Turbo DNase (Ambion) (2 U per 2 μg NA) or RNase A (Invitrogen) (0.4 μg per 2 μg NA) with 2 mM calcium chloride for 30 min at 37°C and analyzed on a denaturing RNA gel. The loading buffer comprised 5× RNA sample buffer {4 mM EDTA, 2.7% formaldehyde, 30.8% formamide, 20% glycerol, 40% 10× MOPS buffer [200 mM 3-(*N*-morpholino)propanesulfonic acid (MOPS), 30 mM sodium acetate trihydrate, 10 mM EDTA; pH adjusted to 7.0 with sodium hydroxide]} diluted to 1×, 25% FORMAzol (Molecular Research Center, Inc.), and 0.05 mg/ml ethidium bromide. After heating for 1 to 3 min at 85°C and cooling to 4°C, samples were immediately loaded onto a 1.2% agarose gel prepared in 1× MOPS buffer with 5% formaldehyde and run at 75 V in 1× MOPS buffer.

### Crystal screening.

Crystallization trials of UL11 constructs at several concentrations (∼12 to 18 mg/ml) were set up using nine screens (Classics Suite [Qiagen], Index [Hampton Research], in-house Harrison Lab Grid Screen, Peg/Ion [Hampton Research], Protein Complex Suite [Qiagen], SaltRx [Hampton Research], Top 96 [Anatrace], Wizard 1-4 [Rigaku]) in 96-well sitting-drop trays with drops containing 0.2 μl protein and 0.2 μl crystallization solution dispensed by Phoenix liquid handling robot (Art Robbins). Crystallization plates were stored at ambient temperature and evaluated using a stereo microscope daily for a week and periodically during subsequent months.

### Phase separation assays.

Macroscopic and microscopic evaluation of UL11 liquid-liquid phase separation (LLPS) was performed using UL11-StII purified as described above in buffer A at various concentrations. In the experiment shown in Fig. 5B and C, UL11-StII was diluted from 18 mg/ml to 9 mg/ml, chilled in a 4°C chill block to induce droplet formation, warmed by hand to clear droplet formation, and either viewed by eye in a tube or with a stereo light microscope as a 2-μl drop on a glass coverslip. The LLPS behavior was also seen at the 18 mg/ml concentration and after one cycle of freezing and thawing. In the experiment shown in Fig. 5D, purified UL11-StII was concentrated to 10 mg/ml, flash frozen, and stored at –80°C. Samples were thawed and diluted to marked concentrations in buffer A with different amounts of NaCl or the addition of molecular crowders (100 mg/ml bovine serum albumin [BSA] or 30% polyethylene glycol 350 monomethylether [PEG 350 MME]). For the LLPS experiment in circular dichroism (CD) buffer (Fig. 6A), UL11-StII was subjected to size exclusion chromatography in buffer A containing no NaCl [50 mM HEPES, 0.5 mM tris(2-carboxyethyl)phosphine (TCEP) (pH 7.5)] before frozen storage and diluted to marked concentrations in CD buffer (20 mM sodium phosphate, 100 mM sodium fluoride [pH 8.0]) after thawing. All samples in the LLPS assay were incubated in a 4°C chill block for 2 h, and aliquots were transferred at 4°C to a 96-well plate and stored for 24 h at either 4°C or ambient temperature before visualization with a stereo light microscope. Similar results were seen after 24-h storage in either condition.

### Circular dichroism.

CD was used to investigate secondary structure content of UL11. Purified, nucleic acid-free UL11-StII in buffer A was exchanged into CD buffer (20 mM sodium phosphate, 100 mM sodium fluoride [pH 8.0]) using a PD SpinTrap G-25 column (GE Healthcare Life Sciences) and diluted to various concentrations. After storage at 4°C, far-UV spectral scans from 185 to 300 nm were taken in a 1-mm cuvette at 20°C on a JASCO J-815 spectropolarimeter, continuously scanning at 50 nm/min with 1-nm bandwidth and 1-s data integration time. Five spectra were collected and averaged, and buffer was subtracted for each sample. Machine data were converted to mean residue ellipticity (MRE) using the equation MRE = θ_mrw_ = (MRW × θ)/(10 × *c* × *l*), where MRW is mean residue weight = molecular mass/(number of amino acids − 1], theta (θ) is ellipticity (millidegrees), *c* is concentration in grams per liter, and *l* is path length (centimeters).

### Limited proteolysis, mass spectrometry, and N-terminal sequencing.

Limited proteolysis was conducted as described previously (88). Briefly, 3 μg purified UL11-StII (at 1.5 mg/ml) was incubated for 1 h at room temperature in buffer A with *N*α-*p*-tosyl-l-lysine chloromethyl ketone (TLCK)-treated chymotrypsin/UL11 at a ratio of 1:2,000, 1,000, 500, 200, 100, 50, or 25; tosylsulfonyl phenylalanyl chloromethyl ketone (TPCK)-treated trypsin/UL11 at a ratio of 1:3,200, 1,600, 800, 400, 200, 100, 50, or 25; proteinase K/UL11 at a ratio of 1:2,000, 1,000, 500, 100, 25, or 5; V8 protease/UL11 at a ratio of 1:2,000, 1,000, 500, 100, 25, or 5. All proteases were from Worthington. Samples were boiled for ∼5 min at 95°C with 1× loading buffer (2% SDS, 4% glycerol, 40 mM Tris [pH 6.8], 2% β-mercaptoethanol) to stop the digest and analyzed by SDS-PAGE. For mass spectrometry analysis, 55 μg (at 1.5 mg/ml) purified UL11-StII was digested with V8 protease at a 1:50 protease-to-protein mass ratio. After an hour, phenylmethanesulfonylfluoride (PMSF) was added to a final concentration of 10 mM to stop the reaction. The samples were analyzed by matrix-assisted laser desorption ionization−time of flight mass spectrometry (MALDI-TOF MS) using a dihydroxybenzoic acid (DHB) matrix on a Voyager DE-PRO instrument at the Tufts University Core Facility. For N-terminal sequencing, 33 μg (at 1.5 mg/ml) purified UL11-StII was incubated with V8-protease at a 1:50 protease/protein ratio. The sample was separated on a 4 to 15% SDS-PAG and transferred to polyvinylidene fluoride (PVDF) membrane in transfer buffer [50 mM 3-(cyclohexylamino)-1-propanesulfonic acid (CAPS) (pH 10.5), 10% methanol] using a semidry transfer apparatus. The membrane was stained in 0.05% Coomassie blue R-250 dissolved in 40% methanol and destained first in 50% methanol and then in water. Bands were cut from the air-dried membrane and subjected to N-terminal sequencing by Edman degradation at the Tufts University Core Facility.

### Cross-linking.

Chemical cross-linking was performed as described previously (86). One hundred picomoles of purified UL11-StII in water was incubated with bis(sulfosuccinimidyl)suberate (BS^3^) (Fisher Scientific) at 0, 1, 2, 4, 8, or 10 molar ratios of cross-linker to protein (1.5 mg/ml) in amber tubes in the dark at room temperature for 20 min. To stop the reaction, 1 M Tris-HCl (pH 8.0) was added to the final concentration of 50 mM and incubated for 15 min. Half of the reaction was loaded onto an SDS-PAG and visualized by Coomassie blue staining.

### Size exclusion chromatography−small angle X-ray scattering.

Small-angle X-ray scattering coupled with size exclusion chromatography (SEC-SAXS) experiments were conducted at the BioSAXS beamline G1 at the Cornell High Energy Synchrotron Source (Ithaca, NY). UL11-StII was centrifuged at 16,000 × *g* for 5 min at 4°C to remove aggregates, and a 100-μl sample was injected onto a Superdex 200 Increase 5/150 (GE Healthcare Life Sciences) in buffer A at 0.3 ml/min and 4°C and fed directly into a proprietary SEC-SAXS flow cell with a glass window. To ensure an adequate signal, the concentration of UL11-StII was ∼18 mg/ml. Samples were irradiated with a 9.9496-keV (1.246122-Å) beam with 5.7 × 10^11^ photons/s flux and diameter of 250 μm by 250 μm, and 2-s images were recorded through the duration of the run on a Pilatus 100K-S detector (Dectris) at a sample-to-detector distance of 1,521.09 mm resulting in a *q* range of 0.006 Å^−1^ < *q* < 0.8 Å^−1^ where scattering vector *q* = 4πsin(θ)/λ. SAXS data were processed using the RAW software package (https://sourceforge.net/projects/bioxtasraw/) (89) by selecting and averaging the scattering curves from buffer data frames (evaluated by integrated scattering intensity correlated with background) subtracting this averaged buffer curve from each subsequent curve and calculating the resulting radius of gyration (*R_g_*), selecting and averaging sample curves with a consistent *R_g_*, and finally subtracting the buffer average curve from the sample average curve. These curves were further analyzed using AUTORG, DATGNOM, AMBIMETER (90), and EOM 2.0 (59) as implemented in RAW and the ATSAS software package (https://www.embl-hamburg.de/biosaxs/software.html) (91). AUTORG was used to define the Guinier region, calculate *R_g_* and *I*(0) parameters, and evaluate uncertainty in these parameters. DATGNOM (as implemented in RAW) was used to generate the pair distance distribution function [*P*(*r*)] from the subtracted experimental data and automatically evaluate it prior to manually adjusting the automatically assigned *D*_max_ value to improve *P*(*r*) shape. AMBIMETER was used to predict whether *ab initio* models generated from the *P*(*r*) would be unique. EOM 2.0 was used to generate a pool of 10,000 macromolecules randomly sampling the conformational space available to the UL11-StII sequence. Theoretical SAXS curves were generated for random combinations of these models, and the subensemble of conformers coexisting in solution that yielded the best fit to the experimental SAXS data was selected. Subensemble selection, or ensemble optimization, was performed three times for the same pool of random models. No symmetry information or structures were specified as constraints in EOM, which allowed for the generation of random configurations of the backbone based only on sequence. *R_g_* and *D*_max_ values calculated from the species in each group were represented in histograms.

### Data availability.

Small-angle scattering data have been deposited into the SASBDB under code SASDEX4.
