# Supplementary material for: Conserved Outer Tegument Component UL11 from Herpes Simplex Virus 1 Is an Intrinsically Disordered, RNA-Binding Protein
Source: mBio. 2020 May 5;11(3):e00810-20. doi: 10.1128/mBio.00810-20 (PMC7403781; doi:10.1128/mBio.00810-20)
Supplement: FIG S1 [file mBio.00810-20-sf001.pdf]

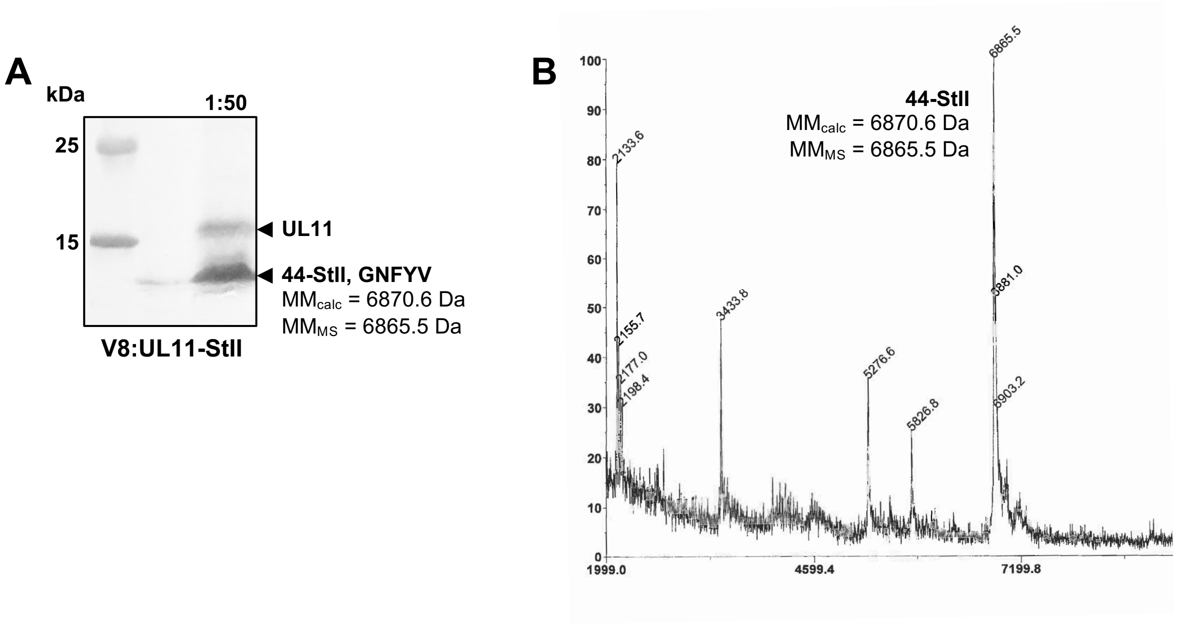

Fig. S1. Identification of V8 proteolytic fragments. (A) Limited proteolysis of UL11-StII with V8 protease. N-terminal sequence of the major proteolytic fragment is shown along with the molecular masses calculated from the sequence (MM_{calc}) and determined by mass spectrometry (MM_{MS}). (B) MALDI-TOF mass spectrometry analysis of the same digest. The x axis shows mass to charge ratio (m/z) in Da and the y axis shows relative abundance (%) scaled for the mass range in x. Molecular mass (MM_{MS}) of the major peak is shown along with the mass calculated from the sequence (MM_{calc}).
