# Supplementary material for: Conserved Outer Tegument Component UL11 from Herpes Simplex Virus 1 Is an Intrinsically Disordered, RNA-Binding Protein
Source: mBio. 2020 May 5;11(3):e00810-20. doi: 10.1128/mBio.00810-20 (PMC7403781; doi:10.1128/mBio.00810-20)
Supplement: TABLE S1 [file mBio.00810-20-st001.pdf]

Table S1. Primers used to generate UL11 constructs.

Primer	Sequence	Description
AK34	5'- AAAGGAAGGGAAGAAAGCGAAA-3'	pET downstream primer, reverse
AK37	5'- ATACCGCGAAAGGTTTTGCG-3'	pET upstream primer, forward
CM121	5'-TAATCGATTACCATGGGCCTCTCGTTCTCC-3'	UL11 N-terminus, forward, NcoI site underlined
CM122	5'-GGGATCTAGTCTCGAGTTATTATTCGCTATCGGACATGGGG-3'	UL11 C-terminus, reverse, XhoI site underlined
CM131	5'-AAATTGTGGGTGGCTCCACTCGCTATCGGACATGGG-3'	UL11-StII, SOE internal reverse
CM132	5'-CACCCACAATTTGAGAAGTAATAACTCGAGCGGCC-3'	UL11-StII, SOE internal forward
CM136	5'-TGGGTGGCTCCAACGCAGGCGCTG-3'	UL11(1-65)-StII, SOE internal reverse
CM137	5'-CAGCGCCTGCGTTGGAGCCACCCA-3'	UL11(1-65)-StII, SOE internal forward
pGEX3	5'-GTCAGAGGTTTTCACCGTCAT-3'	pGEX downstream primer, reverse
pGEX5	5'-ATAGCATGGCCTTTGCAGGGCT-3'	pGEX upstream primer, forward
